# Cobalt Alloy Implant Debris Induces Inflammation and Bone Loss Primarily through Danger Signaling, Not TLR4 Activation: Implications for DAMP-ening Implant Related Inflammation

**DOI:** 10.1371/journal.pone.0160141

**Published:** 2016-07-28

**Authors:** Lauryn Samelko, Stefan Landgraeber, Kyron McAllister, Joshua Jacobs, Nadim James Hallab

**Affiliations:** 1 Department of Orthopedic Surgery, Rush University Medical Center, Chicago, Illinois, United States of America; 2 Department of Immunology, Rush University Medical Center, Chicago, Illinois, United States of America; 3 Department of Orthopaedics, University Hospital Essen, University of Duisburg-Essen, Hufelandstrabe 55, 45122, Essen, Germany; French National Centre for Scientific Research, FRANCE

## Abstract

Cobalt alloy debris has been implicated as causative in the early failure of some designs of current total joint implants. The ability of implant debris to cause excessive inflammation via danger signaling (NLRP3 inflammasome) vs. pathogen associated pattern recognition receptors (e.g. Toll-like receptors; TLRs) remains controversial. Recently, specific non-conserved histidines on human TLR4 have been shown activated by cobalt and nickel ions in solution. However, whether this TLR activation is directly or indirectly an effect of metals or secondary endogenous alarmins (danger-associated molecular patterns, DAMPs) elicited by danger signaling, remains unknown and contentious. Our study indicates that in both a human macrophage cell line (THP-1) and primary human macrophages, as well as an *in vivo* murine model of inflammatory osteolysis, that Cobalt-alloy particle induced NLRP3 inflammasome danger signaling inflammatory responses were highly dominant relative to TLR4 activation, as measured respectively by IL-1β or TNF-α, IL-6, IL-10, tissue histology and quantitative bone loss measurement. Despite the lack of metal binding histidines H456 and H458 in murine TLR4, murine calvaria challenge with Cobalt alloy particles induced significant macrophage driven in vivo inflammation and bone loss inflammatory osteolysis, whereas LPS calvaria challenge alone did not. Additionally, no significant increase (p<0.05) in inflammation and inflammatory bone loss by LPS co-challenge with Cobalt vs. Cobalt alone was evident, even at high levels of LPS (i.e. levels commiserate with hematogenous levels in fatal sepsis, >500pg/mL). Therefore, not only do the results of this investigation support Cobalt alloy danger signaling induced inflammation, but under normal homeostasis low levels of hematogenous PAMPs (<2pg/mL) from Gram-negative bacteria, seem to have negligible contribution to the danger signaling responses elicited by Cobalt alloy metal implant debris. This suggests the unique nature of Cobalt alloy particle bioreactivity is strong enough to illicit danger signaling that secondarily activate concomitant TLR activation, and may in part explain Cobalt particulate associated inflammatory and toxicity-like reactions of specific orthopedic implants.

## Introduction

Recent studies have shown Cobalt alloy implant debris are the central cause of unexpected early failures associated with certain designs of metal-on-metal (MoM) hip joint replacements [1–3]. The pathophysiological reasons for this are different than the “normal” slow inflammatory failure of implants over their expected 15–20 years of use. It is well established that over the long term (>15 years) particulate plastic and metal debris cause implant failure by inducing a subtle but persistent innate macrophage inflammatory responses (i.e. granulomas) that slowly invades the bone-implant interface, leading to bone resorption and eventual painful loosening of the implant [4–7]. This slow inflammatory response is attributed primarily to monocyte/macrophage reactivity to phagocytosed particles. Previous reports show the NLRP3 inflammasome danger signaling pathway plays a central role as a sensor and transducer of stress and danger signals (Danger associated molecular patterns, DAMPs) into inflammatory signals in the cytosol of antigen presenting cells (APCs) after contact with certain non-biological challenge agents such as alum, asbestos and implant debris [6;8–13]. Previous in vitro and in vivo studies of cobalt ions have shown toxicity responses occur at relatively low concentrations <1mM [3;6;14]. Cobalt alloy debris has been established to elicit inflammasome danger signaling, a mechanism central to innate monocytes/macrophage based pro-inflammatory responses (i.e. from initial lysosomal destabilization and NADPH oxidase induction of ROS, to NLRP3-ASC olgiomerization, and Caspase-1 conversion of pro-IL-1β and pro-IL-18 to mature IL-1β and IL-18, respectively) [9;13;15]. However, the relative degree to which PAMP associated Toll-like receptor (TLR) signaling is directly or indirectly capable of potentiating Cobalt related inflammasome mediated inflammation has not been established for this highly reactive type of implant debris.

Prototypical PAMPs produced by Gram-negative (i.e. lipopolysaccaride, LPS) bacteria are recognized by cell surface TLR4 [16]. The role of TLRs in regulating immune reactivity to implant debris remains controversial in general and unknown for particulate Cobalt alloy debris in the submicron to micron size range. Previous studies have shown that soluble (ionic) metal challenge (e.g. Nickel ions) can cause TLR4 activation that is dependent on human TLR4 histidine pocket residues H456 and H458 [17]. Some reports show both Nickel and Cobalt ions challenge can facilitate TLR4 homodimerization independent of MD2 [18] while others show evidence of MD2 dependence in metal ion TLR4 activation [19]. It has also been reported that metal particulate implant debris may be insufficient to activate TLRs [20;21] of macrophages/histiocytes in peri-prosthetic tissues [22;23].

However, these past investigations do not examine the relative activation of inflammasome danger signaling vs. TLR4 activation vs. others, given it has also been shown that the concentrations of metal used in these previous studies (>0.5mM) are potent inflammasome activators [6;24]. To further confuse the issue, recent studies have shown that activation of danger signaling by DAMPs can induce the release of endogenous stress signals (such as High mobility group box 1 protein and heat shock protein 60) that can activate the TLR4 pathway [25–31]. This calls into question whether past reports of metal activated TLR’s do so directly or indirectly through endogenous danger signaling induced stress signals. Thus, distinguishing between which mechanism(s) dominates at clinically relevant challenge concentrations are critical to understanding cobalt induced inflammation/pathogenesis. This distinction is important for mitigating implant debris immune reactivity, given inhibition of PAMP signaling through TLR4 has been put forward as a means to combat debris induced inflammation and aseptic osteolysis [20;21]. However, the relative degree to which interfering with TLR4 activation vs. NLRP3 inflammasome reactivity to inhibit reactivity to implant debris remains unknown. It is unclear if pharmacologic blocking of TLRs would be effective target(s) to mitigate implant debris induced inflammation/osteolysis.

Does TLR activation dominate Cobalt-alloy particulate induced inflammation/pathology as compared to NLRP3 inflammasome? To determine if TLR activation due to Cobalt alloy particles dominate subsequent inflammation and inflammatory bone loss, clinically relevant Cobalt alloy particulate debris either alone or combined with LPS (TLR4 ligand) were used as challenge agents with: 1) a human monocyte/macrophage cell line (THP-1), 2) primary human monocytes/macrophages and 3) an established mouse calvaria model of inflammatory bone loss (murine TLR4 lacks human histidine residues associated with soluble metal -TLR binding [17]). Cobalt alloy and LPS testing was conducted under both normal, inflammasome inhibited and TLR4 inhibited conditions (i.e. neutralizing antibodies to TLR4), while measuring downstream DAMP and PAMP inflammation in vitro (IL-1β and TNF-α) and inflammatory bone loss in vivo.

## Materials and Methods

### Ethics Statement

This study and consent process received approval from the Rush University Institutional Review Board (10052606-IRB01-CR02). Participants provided their written consent to participate in this study. All participants were 18 years of age or older.

Animal Research: C57BL/6 male mice (12 weeks old) were purchased from the Jackson Laboratory (Bar Harbor, Maine) and kept under pathogen-free conditions. All experiments were carried out under the guidelines of the Institutional Animal Care and Use committee at Rush University Medical Center. The protocol was approved by the committee on the Ethics of Animal Experiments of Rush University Medical Center (IACUC Number: 13–065) and all in our study was conducted adhering to the institution’s guidelines for animal husbandry, and followed the guidelines and basic principals in the Public Health Service Policy on Humane Care and Use of Laboratory Animals, and the Guide for the Care and Use of Laboratory Animals, United States Institute of Laboratory Animal Resources, National Research Council. All efforts were made to minimize suffering; all manipulations were performed under medetomidine/ketamine anesthesia and mice were sacrificed by cervical dislocation after anesthesia.

### Media and Challenge Agents

Human monocyte cell line THP-1 (ATCC) were cultured with RPMI 1640 supplemented with L-Glutamine, Penicillin, Streptomycin, 25 mM Hepes (Lonza, Walkersville, MD USA) and 10% heat inactivated fetal bovine serum (FBS; Hyclone Laboratories, Logan, UT). Human primary monocytes/macrophages were cultured in RPMI 1640 supplemented with L-Glutamine, Penicillin, Streptomycin, 25 mM Hepes and 10% heat inactivated autologous serum.

LPS (TLR4 positive control), PAb Control (control isotype) and PAb-hTLR4 (polyclonal antibody to human TLR4), Z-VAD-FMK (inhibitor of caspase-1 activation in NLRP3 induced cells) (InvivoGen), and selective lysosomal Cathepsin-B inhibitor (CA-074-Me) (Sigma-Aldrich) were used in challenged monocytes/macrophages. The mean particle sizes of Cobalt-alloy (Cobalt-alloy, approx 60%Co, 28%Cr, <6%Molybdenum, <1%Nickel, ASTM F75) particles were characterized by using low angle laser light scattering (LALLS) and Scanning Electron Microscopy (SEM). Cobalt-alloy particles had a mean diameter of 0.88 μm diameter ECD (>95% less than 2μm, >80% less than 1 μm, range 0.2–11μm diameter ECD, BioEngineering Solutions Inc., Oak Park, IL). This size of particulate debris has been shown to be clinically relevant and able to induce inflammatory responses in innate immune cells [6;32]. Subsequent to characterization, particles were cleaned, sterilized, and tested for endotoxin levels before use in experiments (<0.01 eU, Kinetic QCL, Bioengineering Solutions Inc, Oak Park, IL). Challenge concentrations were previously determined to be non-toxic after 24 hours of challenge [6;33].

### Cell Purification

Blood samples were obtained intravenously from healthy volunteers (n = 5) under Rush University IRB-approved informed consent. Peripheral blood mononuclear cells (PBMCs) were isolated from heparinized whole blood from donors by Ficol gradient separation. Human peripheral blood CD14+ monocytes were subsequently purified from the collected buffy coats (PBMCs fraction) using EasySep magnet according to the manufacturer’s instructions for EasySep Human CD14 Positive Selection Kit (Stemcell Technologies Cat. Number 18058).

### Cell Culture and Blocking Experiments

Trypan Blue Stain exclusion method was performed to determine cell viability over 90% for all experiments and cells were subsequently plated in 48-well plates/0.5mL/well. Human THP-1 monocytes and isolated human primary monocytes were differentiated into macrophages by culturing 2.0×10^5^ monocytes in 48 well plates with either phorbol ester (TPA 200nM) or MCSF (50ng/mL) respectively, for 18–24 hours and then washed and rested for 24 hours prior to metal challenge (as determined previously to yield maximal inflammasome monocyte/macrophage responses)[6;33]. Newly differentiated macrophages were challenged with TLR4 LPS and/or with cobalt-alloy particles for 20 hours at 37°C 5% CO_2_. Supernatants were subsequently analyzed for mature IL-1β, TNF-α, IL-6 and IL-10 production. PAb Control (proper control for use with PAb-hTLR4), PAb-hTLR4 at 4 ug/mL were added with THP-1 cells and human primary monocytes/macrophages one hour prior to challenge as a control isotype or to neutralize human TLR4-induced cellular activation, respectively. Cathepsin-B inhibitor (10uM) or ZVAD-FMK (20uM) was added to THP-1 cells or human primary monocytes/macrophages one hour prior to challenge to block the effects of cytosolic lysosomal Cathepsin-B on NLRP3 activation or caspase-1 activation respectively [11;24].

Mouse cell culture: Thioglycollate-induced peritoneal macrophages were obtained 4 days after an intraperitoneal injection of 1 ml 3% thioglycollate medium in male wild- type C57BL/6. Cells were harvested by flushing the peritoneal cavity with 10 ml ice-cold sterile PBS. Cells were seeded on tissue culture plates at a density of 1X10^6^ cells/well in 24-wells plates. Cells were cultured in complete RPMI-1640 medium. After 3 h adhesion, non-adherent cells were removed and the remaining peritoneal macrophages were analyzed for cytokine production after 24 h of challenge agents (performed in triplicate).

### ELISA

Sandwich ELISAs for human IL-1β, TNF-α, IL-6 and IL-10 were used to detect challenged THP-1 and human primary cells using manufacturer’s instructions (R&D systems). Sandwich ELISAs for mouse IL-1β and TNF-α were used to detect challenged isolated peritoneal macrophages using manufacturer’s instructions (R&D systems). Graphical data is shown as the mean concentration of cytokines (+SEM) of three independent experiments, each performed in triplicate.

### Confocal Microscopy

THP-1 monocytes (ATCC) were cultured in RPMI-1640 10% fetal bovine serum (FBS) (Hyclone Laboratories, Inc) and differentiated into macrophages by culturing 5.0 × 10^5^ in glass chamber slide with phorbol ester (TPA) for 18–24 hrs. Differentiated THP-1 macrophages challenged with Cobalt alloy particles and incubated at 37°C for 4 hrs with 15μg/ml DQ Ovalbumin (Invitrogen) and were subsequently fixed with histochoice, washed with PBS, mounted (vectasheild) prior to confocal imaging (Zeiss LSM 510, 488nm).

### Calvarial Osteolysis Model

All in vivo murine studies performed were approved by Rush University Institutional Animal Care Committee. Wild type male mice C57BL/6 were obtained from Jackson Laboratories (Bar Harbor, ME). 12 week old male C57BL/6 mice were shaved prior to calvaria surgery, and the area was sterilized with 70% ethanol and iodine. Mice were then treated with one of 4 regimens (a) sham-surgery (sterile PBS), (b) 5 μg/mL LPS TLR4 agonist, (c) Cobalt-alloy particles at a dose 2 mg/mouse calvaria (d) Cobalt-alloy particles + LPS. Calvaria were dosed with 2 mg of Cobalt-alloy particles by making a midline sagittal incision over the calvaria, exposing the intact periosteum. MicroCT and Osteolysis Analysis: 3D-CT was performed with an isometric resolution of 9 um. Areal osteolysis was determined at 10 days post-op by scanning isolated calvaria in a Scanco40 (ScanCo Medical, Basserdorf, Switzerland) transferred to an Amira 5.2 (TGS, Mercury Computer Systems, Inc., San Diego, CA) then image analyzed using ImageJ for % osteolysis within a circular control volume of approx 1cm located medially (reported as the means of n = 5 mice per group with SEM of one individual experiment).

### Tissue Histology

Wild-type calvaria were removed and fixed in 4% paraformaldehyde for 48 hours, followed by decalcifying in 10% EDTA for 4 days and paraffin embedding. Sections (5 mm) were cut and H&E staining was performed. Photomicrographs were taken at an original magnification of ×100 or ×400. Inflammatory infiltration in midsaggital suture areas was quantified from five images per animal with SigmaScan Pro Image analysis version 5.0.0 software.

Four-micrometer thick sections of calvaria were collected at the depth at which the presence of particles was detected within the calvarial tissue. The sections were mounted on glass slides and subsequently HE staining was performed. HE-stained specimens were photographed digitally using a standard high-quality light microscope (Leica, Wetzlar, Germany). The image was oriented with the midline suture in the middle of the field. The sections were coded and blinded prior to analysis. Histomorphometric measurements were performed with the image analysis software Aperio® (LeicaTM, Wetzlar, Germany).

### Statistical Analysis

Comparison of two groups was performed using Students *t* test. The comparison of more than two data sets was performed using one-way ANOVA, using the Prism 6.0 program (GraphPad, San Diego, CA). Statistical difference was considered significant at p < 0.05. Note: */** in graphical figures represents statistical significance of challenged group to respective control.

## Results

### TLR4 agonist and Cobalt-alloy particles induce THP-1 macrophage dose responses of IL-1β and TNF-α

**Fig 1:**
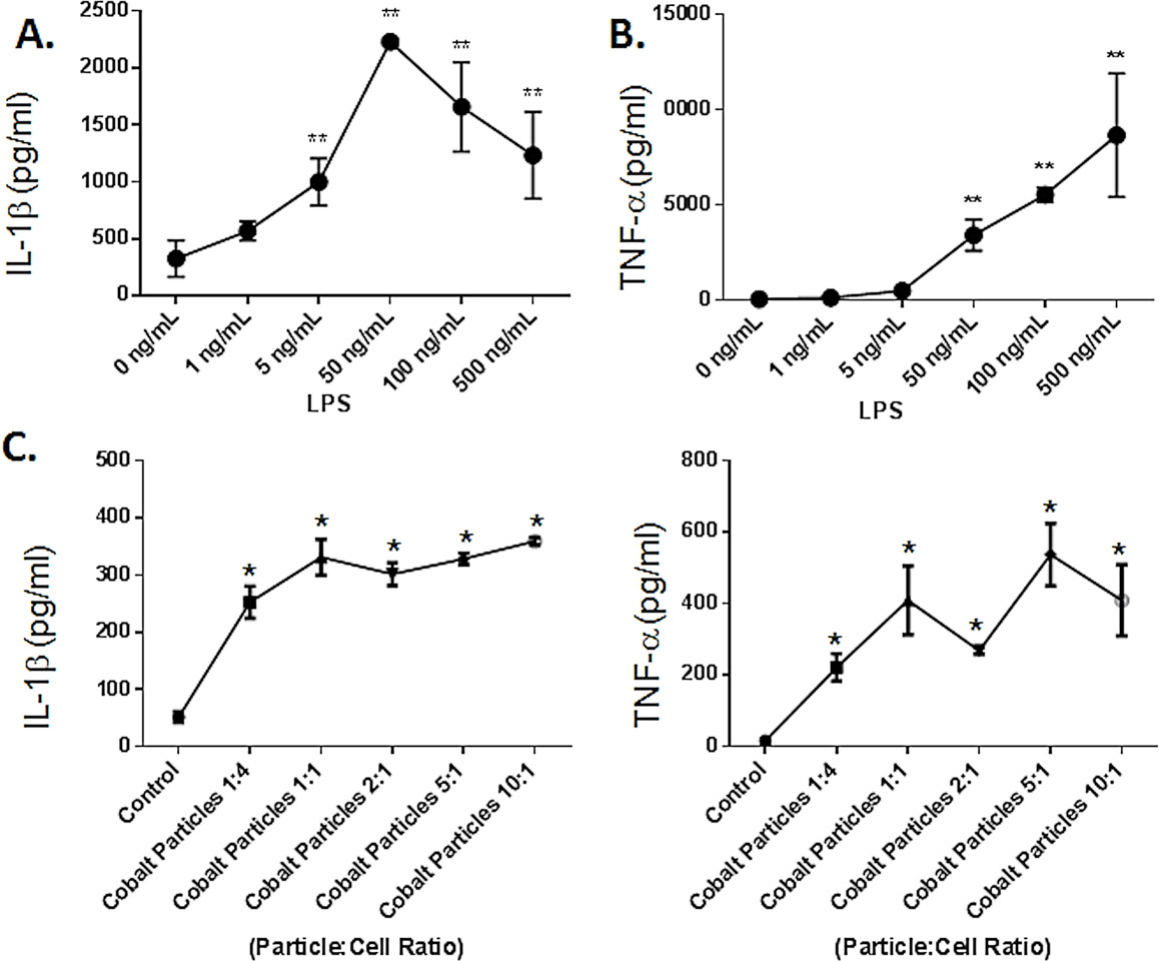
TLR4 LPS and Cobalt-alloy particles induce THP-1 macrophage secretion of IL-1β and TNF-α in a dose dependant manner. IL-1β and TNF-α cytokine production was assessed after THP-1 differentiated macrophages were challenged with (A-B) increasing concentrations of TLR4 agonist LPS and (C-D) increasing dose of Cobalt-alloy (particles to cells ratio) for 20 h and was quantified by ELISA. Cobalt induced significantly less IL-1β and TNF-α than TLR agonist LPS. Note: * indicates *p*<0.05 respective to control and ** represents significance at p<0.01 respective to 0ng/mL.

Dose responses were assessed using a human macrophage (THP-1) cell line with increasing concentrations of LPS and Cobalt-alloy particles (increasing particle:cell ratio). Both IL-1β and TNF-α secretion were measured 20 hours post exposure. Both challenge agents induced concentration-dependent secretion of IL-1β and TNF-α in THP-1 cells (Fig 1). Although IL-1β secretion in response to TLR4 ligand LPS, demonstrated maximal response at 50ng/mL (Fig 1A). While, TNF-α response to LPS challenge was the most significant at the highest concentration 500ng/mL (Fig 1B). All further in vitro experiments using TLR4 ligand LPS were performed however, at standard concentration of 100ng/mL.

A similar concentration-dependent response was observed for Cobalt-alloy induced IL-1β and TNF-α production in THP-1 macrophages (Fig 1C and 1D). TNF-α secretion however, peaked at a dose of 5:1 (particle:cell). Accordingly, all further in vitro experiments were performed with this specific Cobalt-alloy particle to cell ratio. Cobalt-alloy particles independent of PAMP challenge were able to induce macrophage activation (i.e. without addition of LPS) and doses of particles greater than 5 particles per cell induced less responses, consistent with previously reported toxicity responses [6;33]. However, Cobalt alloy particle induced inflammatory responses as determined by IL-1β and TNF-α secretion in vitro were not as pro-inflammatory relative to TLR4 PAMP agonist despite high levels of PAMP agonist (LPS) used at levels corresponding to sepsis (>500pg/mL) [34;35] (Fig 1). This demonstrates that a distinct TLR agonist (LPS) as well as sterile Cobalt-alloy particles can each independently trigger pro-inflammatory cytokine release in THP-1 human macrophages in a dose response manner.

TLR4 agonist LPS and Cobalt-alloy particles in THP-1 Macrophages induce significantly elevated secretion of IL-1β, TNF-α and IL-10, while only Cobalt particles significantly increase IL-1β without concomitant IL-10 increase.

**Fig 2:**
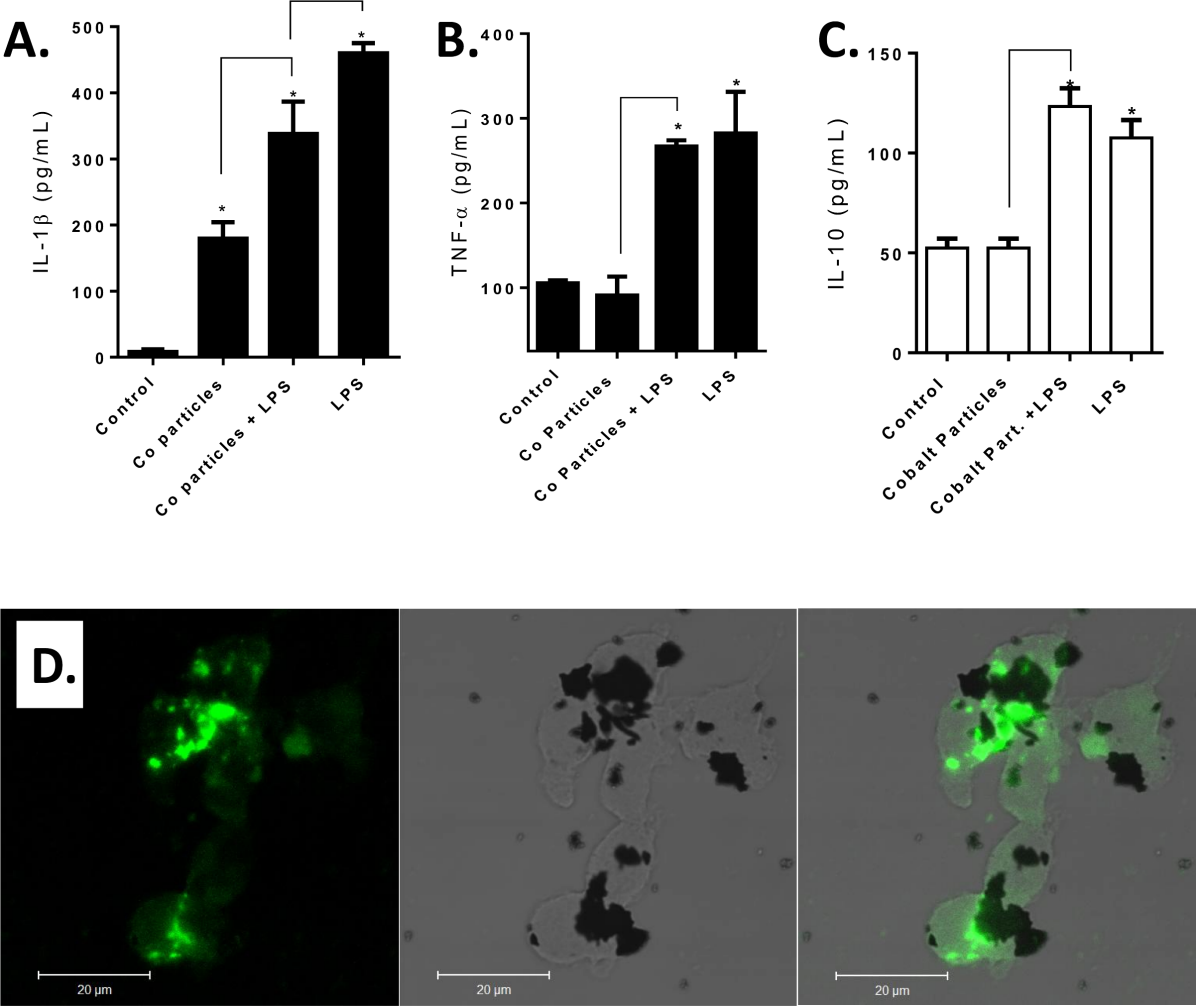
TLR4 agonist LPS and Cobalt-alloy particles in THP-1 Macrophages induce significantly elevated secretion of IL-1β, TNF-α and IL-10, while only Cobalt particles significantly increase IL-1β without concomitant IL-10 increase. (A) IL-1β (B) TNF-α **(C)** and IL-10 cytokine production was assessed after THP-1 differentiated macrophages were challenged with Cobalt-alloy particles (particles:cell = 5:1), Cobalt-alloy/LPS+, and LPS (100ng/mL) for 20 h and was quantified by ELISA. **(D)** THP-1 macrophages were challenged with Co-alloy particles and incubated with 15μg/ml DQ ovalbumin simultaneously for 4 hrs and subsequently were fixed and evaluated for the presence of large pools of dispersed DQ ovalbumin fluorescence co-localized with particles, indicative of lysosomal destabilization. Note: * p<0.05 compared to control macrophages.

THP-1 differentiated macrophages were challenged with Cobalt-alloy particles alone (particles:cell = 5:1), Cobalt-alloy/LPS+ and LPS (100ng/mL) for 20 hours. All challenge agents significantly increased inflammasome mediated IL-1β levels in THP-1 macrophages (**Fig 2A**). However, Cobalt alloy did not significantly increase TNF-α production (**Fig 2B**). The dual challenge of Cobalt-alloy/LPS+ significantly increased both IL-1β and TNF-α production above that of Cobalt-alloy implant debris alone, but was not significantly above LPS alone (100ng/mL). IL-10 was significantly increased to both Cobalt-alloy/LPS+ and LPS (**Fig 2C**). In contrast, Cobalt particle challenge did not significantly affect IL-10 release. Inflammasome activation by Cobalt alloy particles due to lysosomal destabilization was supported when observed in THP-1 macrophages challenged for 4 hrs with Cobalt particles and incubated with DQ ovalbumin simultaneously. Cobalt alloy particles in THP-1 cells demonstrated lysosomal destabilization as indicated by large diffuse pools of DQ ovalbumin fluorescence co-localized with Cobalt alloy particles (**Fig 2D**). This data shows that phagocytosed Cobalt particles resulted in lysosomal destabilization, that in turn likely activates the inflammasome, as demonstrated by the significant increase of IL-1β levels, consistent with previous reports [11;24]. These data support the contention that Cobalt-alloy particles alone induce an immune response via NLRP3 inflammasome dependent mechanism, not TLR4 signaling pathway, unless complexed with LPS.

### Blocking inflammasome danger signaling was more effective than attempts to block TLR4 in both THP-1 human monocytes and macrophages challenged with Cobalt-alloy particles

**Fig 3:**
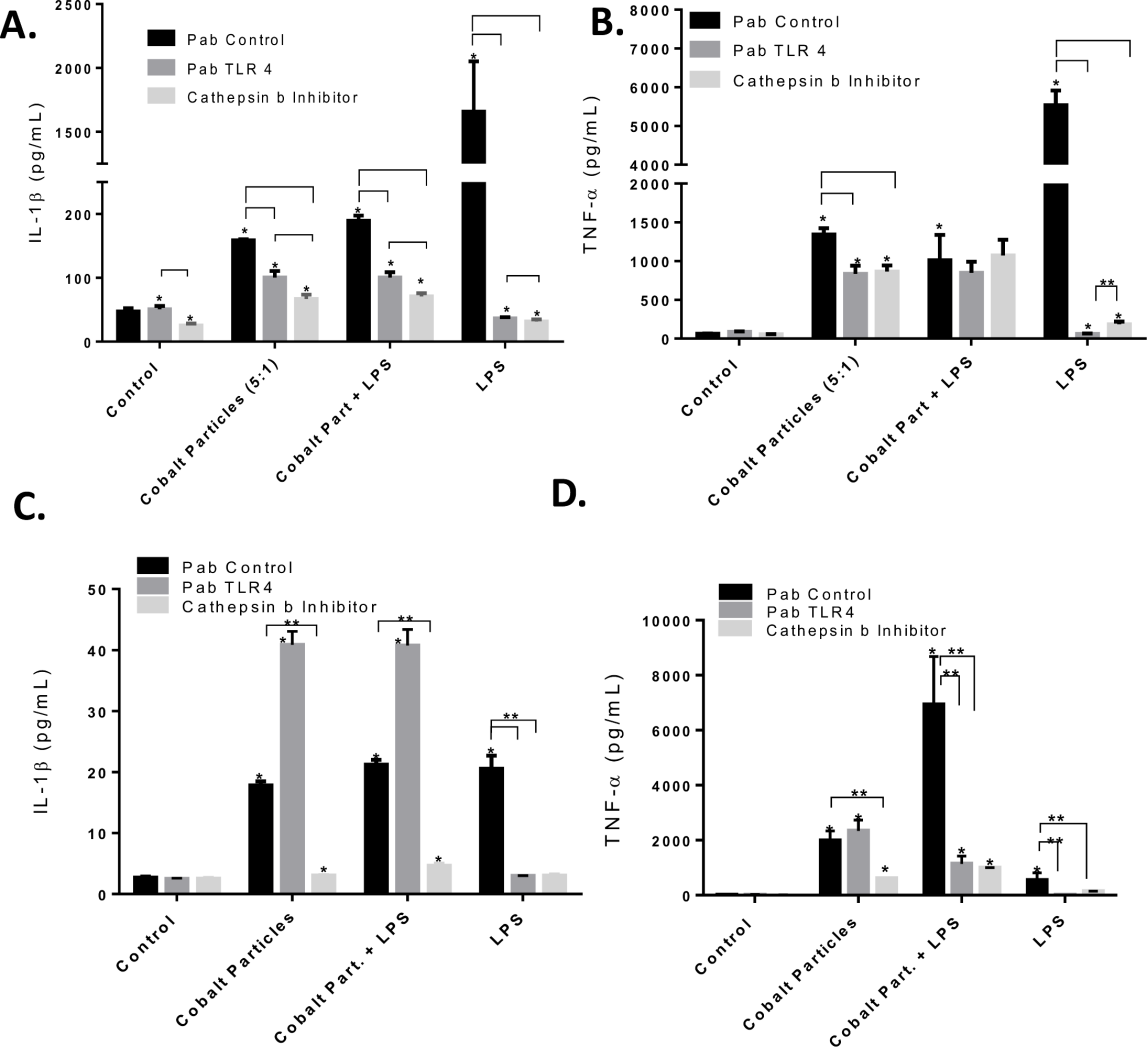
Blocking inflammasome danger signaling was more effective than attempts to block TLR4 in both THP-1 human monocytes and differentiated macrophages challenged with Cobalt-alloy particles. IL-1β and TNF-α was assessed after (A-B) THP-1 differentiated macrophages and (C-D) THP-1 monocytes were challenged with Cobalt-alloy particles (particles:cell = 5:1), Cobalt-alloy/LPS+, and LPS in the presence of either PAb isotype matched control antibody (4μg/mL), PAb-hTLR4 (4μg/mL), or Cathepsin-B inhibitor (10uM) for 20 hours. Cathepsin B danger signal (inflammasome) blocking for Cobalt alloy [6] significantly decreased IL-1β responses to all challenges, more than TLR blocking in both macrophages (A-B) and monocytes (C-D). Note: */** p<0.05 to each treatment groups respective control values

Endotoxin free Cobalt-alloy particles and Cobalt-alloy particles complexed with TLR4 LPS (Cobalt-alloy/LPS+) significantly induced IL-1β and TNF-α secretion in both THP-1 differentiated macrophages (**Fig 3A and 3B**) and monocytes (**Fig 3C and 3D**) when compared with isotype control antibody challenge (PAb control;*p<0.05). Because Cobalt ions have been shown to disrupt maturation of monocytes[36], a total of 48 hours of differentiation was used (24 hrs with TPA and 24 hours rest) prior to addition of metal challenge. A known potent inflammasome inhibitor, Cathepsin-B inhibitor significantly decreased IL-1β (*p<0.05 compared to respective PAb control and PAb TLR4 treated THP-1 cells; **Fig 3A and 3C**) in response to Cobalt alloy challenge agents. Moreover, the Cathepsin-B inhibitor was significantly more effective in attenuating IL-1β to Cobalt-alloy and Cobalt-alloy/LPS+ challenge compared to PAb TLR4 treated THP-1 human macrophages (Fig 3A). In contrast to IL-1β production, TNF-α increases were relatively unaffected by blocking with Cathepsin-B inhibitor or PAb TLR4 for Cobalt or Cobalt/LPS+ treated cells (Fig 3B), suggesting Cobalt-induced endogenous danger signals may activate a TLR4 related NFκB pathway without extracellular TLR4 activation or, alternatively, that Cobalt may activate TLR4 receptors in a manner circumventing PAb TLR4 blocking. However, this latter case is less likely given the significantly increased TNF-α response to LPS over that of Cobalt alloy alone or Cobalt/LPS+ and is less likely given the result of similar testing in undifferentiated monocytes.

Undifferentiated THP-1 monocytes produced significantly increased IL-1β and TNF-α production in response to all challenge agents (Fig 3C and 3D). Surprisingly, PAb TLR4 treatment augmented IL-1β responses of monocytes to all challenge agents except LPS alone (i.e. Cobalt-alloy particles and Cobalt-alloy/LPS+; Fig 3C). It is not known why the PAbTLR4 acted to increase IL-1β and not TNF-α when Cobalt-challenged alone or with LPS. THP-1 monocytes do not readily possess phagocytosis abilities, especially when compared with differentiated THP-1 cells[37]. The Cobalt-PAbTLR4 stimulatory effect was limited to danger signaling type activity (IL-1β) and not TLR related NFĸB/TNF-α. There are several possibilities for this. It may be related to 1) changes in altered propensity for lysosome destabilization/phagocytosis (i.e. ability to internalize particles), or 2) could be due to the ease with which stored IL-1β in monocyte vesicles can be released or 3) could be related to a more complex ability of metals to bind PAbTLR and gain entry to the cell. These possibilities are speculative and require further investigation that is currently beyond the scope of this work.

Cobalt induced endogenous DAMP activation of the TLR4 related NFκB pathway, was supported by Cathepsin-B inhibitor strongly decreasing both IL-1β and TNF-α secretion (Fig 3C and 3D) to Cobalt-alloy particles and Cobalt-alloy/LPS+ challenge (compared to PAb TLR4). Overall, THP-1 monocytes were a magnitude less inflammatory compared differentiated THP-1 macrophages at 20 hours (as assessed by only IL-1β levels, Fig 3). While LPS induced TNF-α release was an order of magnitude greater in differentiated THP-1s when compared to THP monocytes. This was not the case for Cobalt induced TNF-α (where levels were not differentiation dependent) and thus, suggests a different mechanism of TNF-α release than LPS (TLR4). Collectively, these data indicate that innate monocyte/macrophage reactivity to implant debris is dominated by inflammasome activation as evidenced by the potent inhibitory effect of Cathepsin-B on IL-1β production. This argument is strengthened when comparing the relative inhibition of Cathepsin-B to neutralizing antibodies to TLR4 (despite the possibility of in-efficacious blocking of TLR4 to Cobalt alloy via PAb TLR4). Moreover, endotoxin free particles were sufficient for THP-1 macrophage activation alone or when co-challenged with PAMP ligand of TLR4.

### Biological responses to Cobalt-alloy particles in human primary differentiated macrophages induce significantly elevated secretion of both pro and anti-inflammatory cytokines, only when combined with LPS

**Fig 4:**
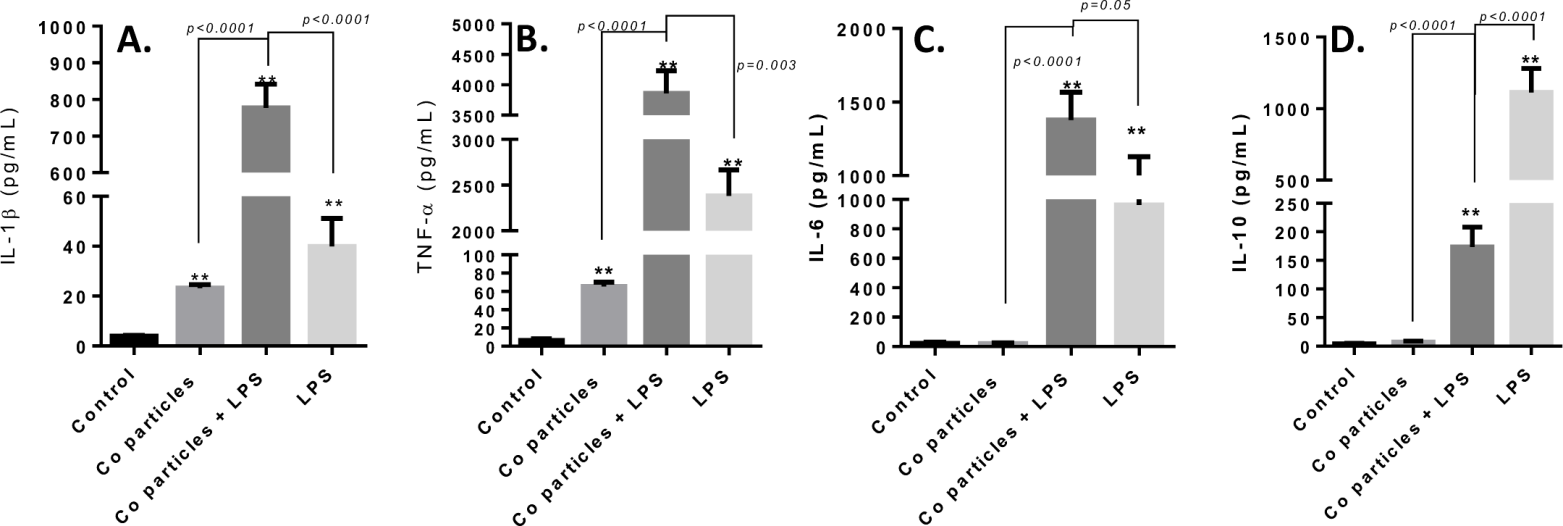
Biological responses to Cobalt-alloy particles in human primary differentiated macrophages induce significantly elevated secretion of both pro and anti-inflammatory cytokines, only when combined with known TLR4 agonist LPS. (A) IL-1β (B) TNF-α (C) IL-6 and (D) IL-10 cytokine production was assessed after human primary differentiated macrophages (n = 4) were challenged for 20 h with Cobalt-alloy particles (particles:cell = 5:1), Cobalt-alloy/LPS+, and LPS. Note: ** p<0.05 to each treatment groups respective control values.

We challenged human primary differentiated macrophages (n = 4) for 20 h and subsequently measured protein secretion of IL-1β, TNF-α, IL-6 and a key anti-inflammatory cytokine IL-10 (**Fig 4**). Both IL-1β and TNF-α were significantly elevated in response to only Cobalt-alloy particles (**Fig 4A and 4B**). In contrast, IL-6 and IL-10 were non-significantly increased, with levels similar to that of control (non-stimulated cells; **Fig 4C and 4D**). While the greatest increase in pro-inflammatory cytokines IL-1β, TNF-α and IL-6 was observed in response to the combined challenge of Cobalt particles with TLR4 LPS, Cobalt-alloy/LPS+, demonstrating a synergistic effect (i.e. greater than additive) of this dual challenge. However, only LPS challenge alone induced the anti-inflammatory cytokine IL-10, even over combined Cobalt-alloy/LPS+ human primary macrophages (Fig 4D). These results demonstrate that pleiotropic responses of Cobalt-alloy particles (vs. TLR4 agonist LPS) that significantly induce both IL-1β and TNF-α in human primary macrophages, but fail to induce NFκB dependent IL-6 and IL-10. The combined indication of synergistic responses when Cobalt particles and LPS are combined along with the absence of IL-10 upon Cobalt only exposure, both support a differential mechanism of activation and provide further support for separate inflammasome dominated activation by Cobalt implant debris (NLRP3 inflammasome vs. TLR4 pathway).

### Blocking TLR4 does not significantly decrease IL-1β and TNF-α response of human primary monocyte/macrophages to Cobalt-alloy particles

**Fig 5:**
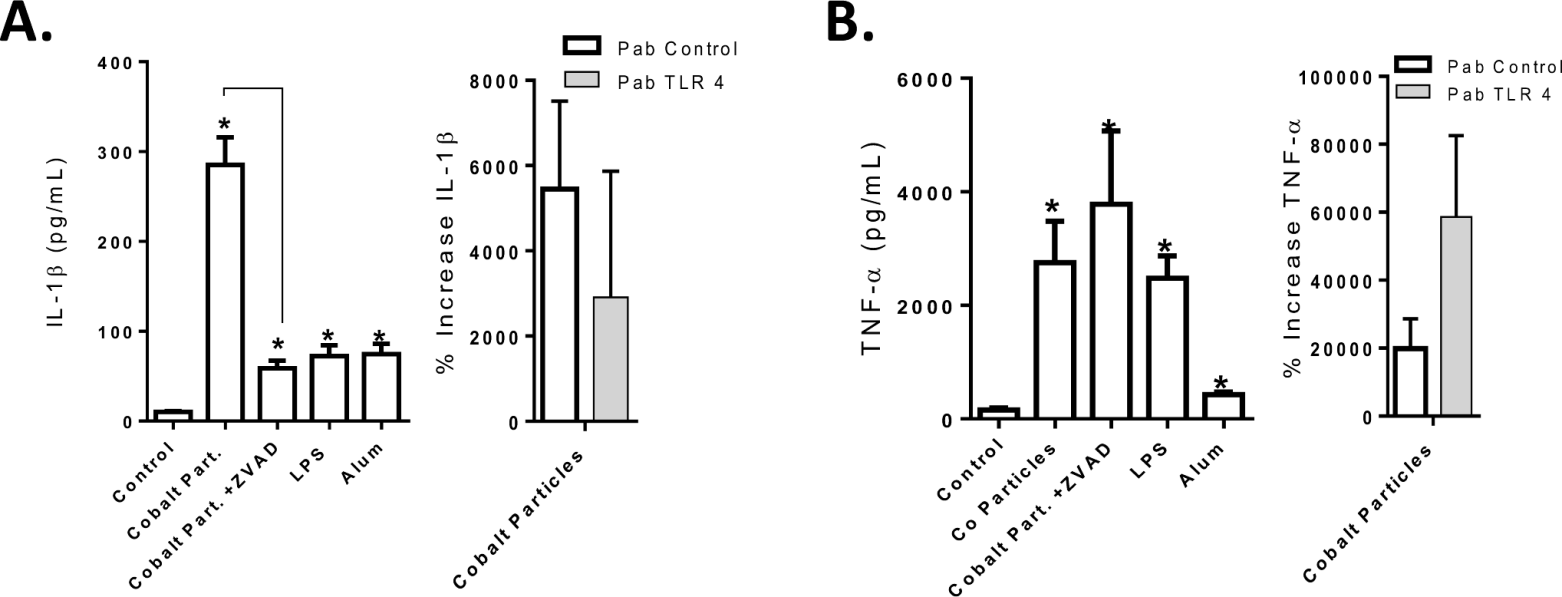
Blocking TLR4 does not significantly decrease IL-1β and TNF-α response of human primary monocyte/macrophages to Cobalt-alloy particles. (A) IL-1β and (B) TNF-α secreted by human primary monocytes/macrophages (n = 5) challenged with Cobalt-alloy particles (particles:cell = 5:1), LPS or Alum (NLRP3 inflammasome activator) for 20 h, with or without ZVAD-FMK (caspase-1 inhibitor) or PAb TLR4 antibody and was quantified by ELISA. Cytokine levels with use of PAb TLR4 are represented as percent increase as compared to respective control cells and averaged as a group. Note: * *p<0*.*05* to each treatment groups respective control values.

TLR activation has been previously shown to prime the activation of NLRP3 through the induction of NLRP3 expression in macrophages [38–40]. Thus, if Cobalt alloy particles were activating both NLRP3 and TLR4, inhibition of TLR4 would likely dramatically decrease IL-1β production, whereas the downstream inhibitor of inflammasome activation ZVAD-FMK significantly reduced IL-1β levels by 80% (Fig 5A). The addition of PAb TLR4 non-significantly reduced the levels of IL-1β response to Cobalt-alloy (Fig 5A). However, the addition of TLR4 neutralizing antibody failed to reduce TNF-α production to Cobalt-alloy particles alone, instead, TNF-α levels were augmented (Fig 5B); indicating down-stream blocking of Cobalt alloy induced lysosomal destabilization etc and subsequent danger signaling may not effectively mitigate endogenous danger signaling of NFκB activity and subsequent TNF-α production.

### An in vivo murine calvarial model of particle-induced osteolysis demonstrates Cobalt-alloy particulate can induce significant inflammatory bone loss, independent of TLR4 activation

**Fig 6:**
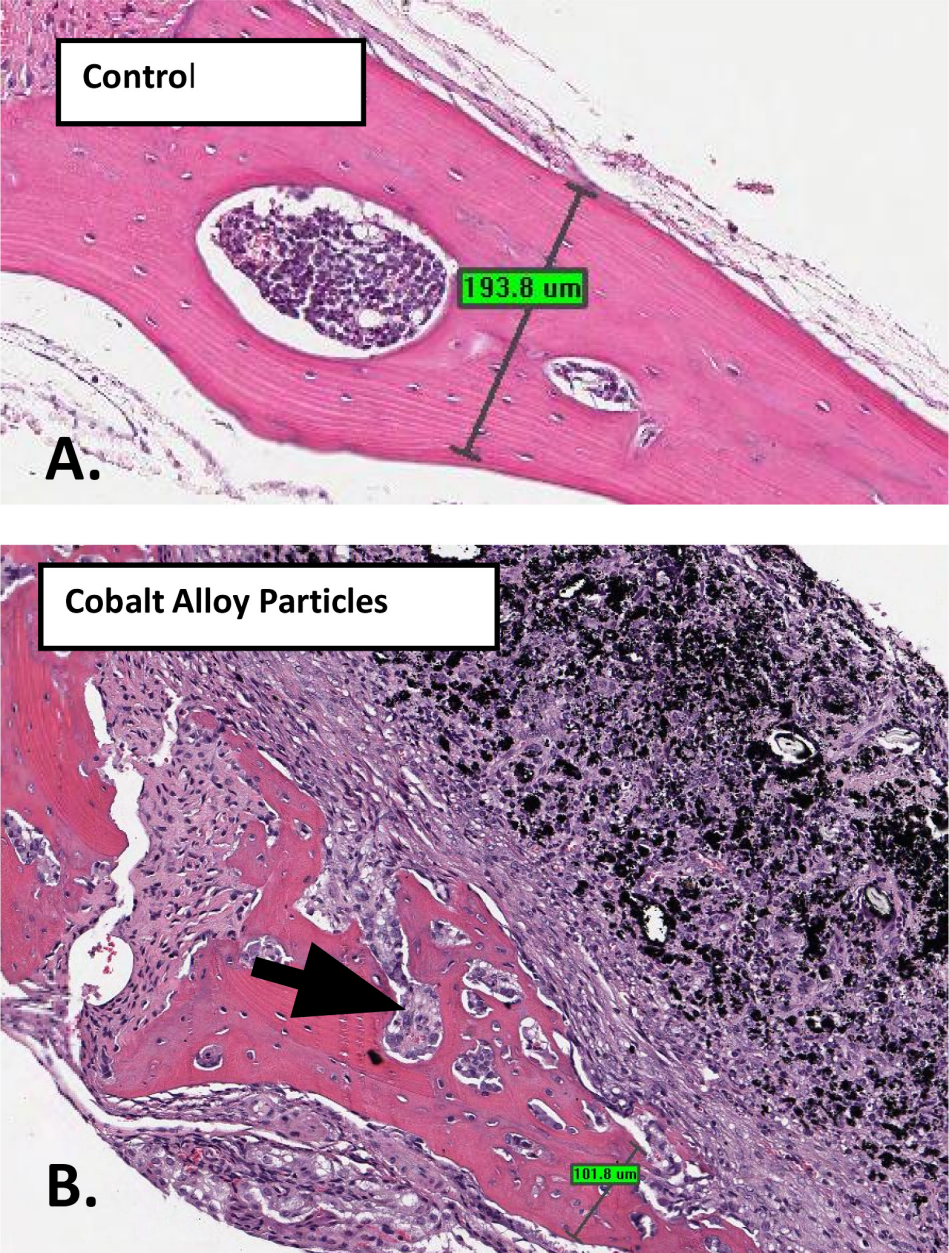
Hematoxylin and eosin staining of C57BL/6 mouse calvarial tissue and bone thickness 10 d post-op that either received (n = 1/5 represented per group): (A) sham-surgery (sterile PBS), showing no signs of inflammation or significant osteolysis with remaining bone thickness at 193.8 um or (B) 2 mg/mouse calvaria of endotoxin-free Cobalt-alloy particles, with inflammatory infiltrate into the calvarial bone identified by arrows and osteolysis with remaining bone thickness at 101.8 um.

**Fig 7:**
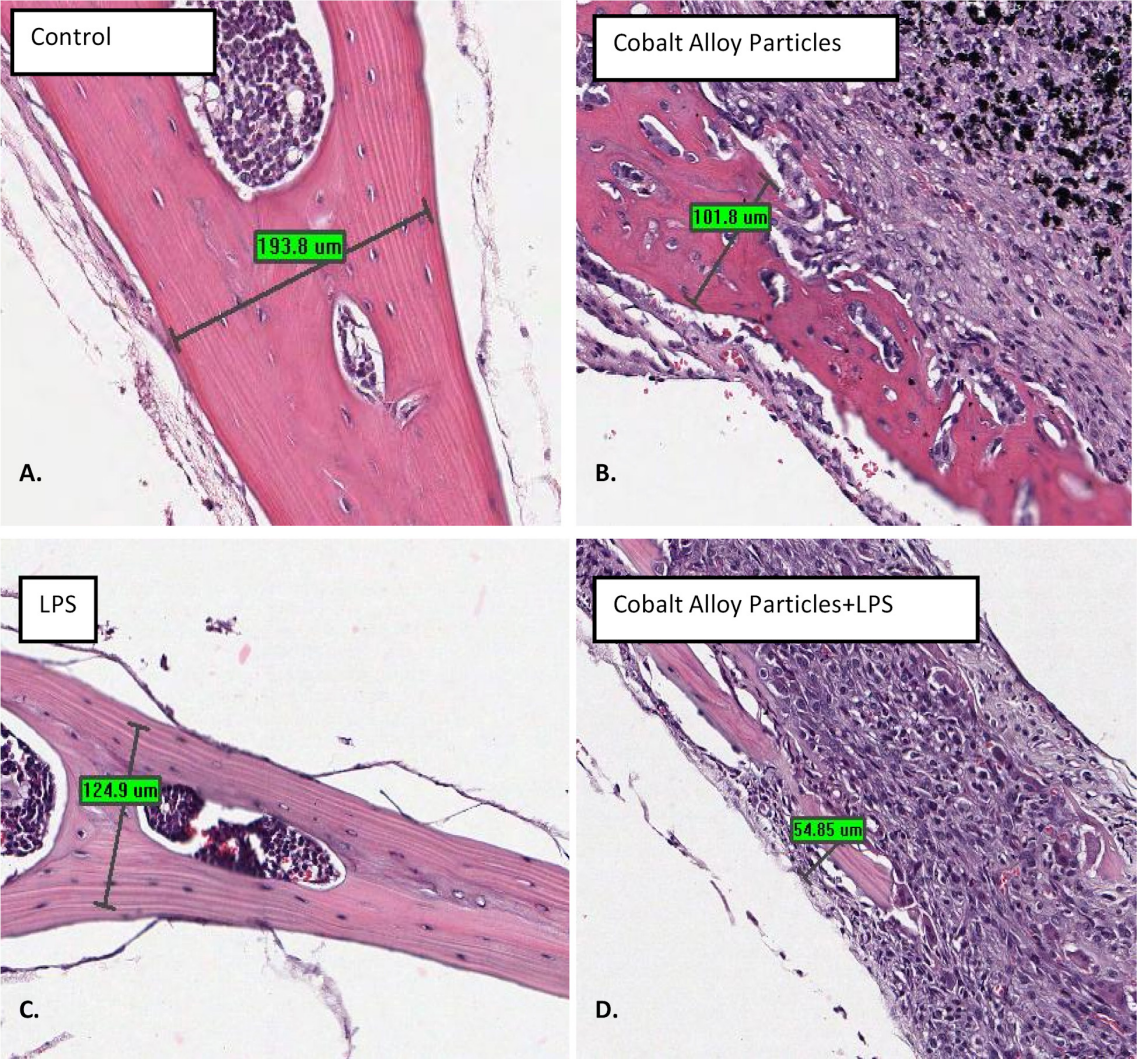
Hematoxylin and eosin staining of C57BL/6 mouse calvarial tissue and bone thickness 10 d post-op that either received (n = 1/5 represented per group): (A) sham-surgery (sterile PBS), (B) 2 mg/mouse calvaria of endotoxin-free Cobalt-alloy particles, (C) 5 μg/mL LPS or (D) Cobalt-alloy/LPS+. Measurements represent remaining bone thickness.

**Fig 8:**
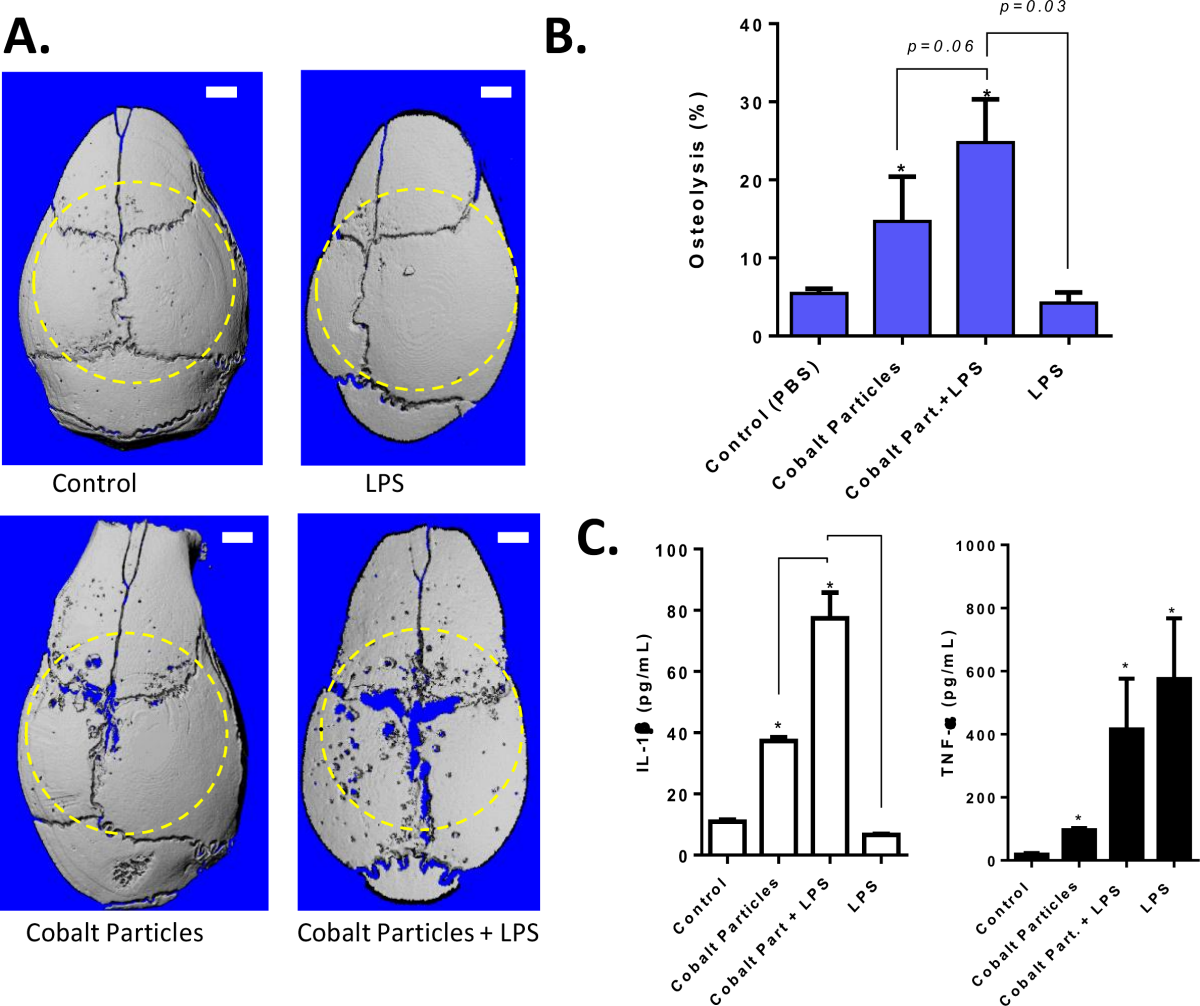
An in vivo murine calvarial model of particle-induced osteolysis demonstrates Cobalt-alloy debris co-challenge with TLR4 induces osteolysis but not more than Cobalt alloy alone. C57BL/6 12 wk old male mice either received (n = 5 per group): (1) sham-surgery (sterile PBS), (2) 2 mg/mouse calvaria of endotoxin-free Cobalt-alloy particles, (3) 5 μg/mL LPS or (4) Cobalt-alloy/LPS+. 10 days later, calvaria were retrieved and analyzed by microCT. (A) Representative images (n = 1/5) and (B) Graphical representation of the percentage decrease in bone thickness relative to sham controls (average of n = 5 per group). (C) IL-1β and TNF-α cytokine production by isolated peritoneal male C57BL/6 macrophages after 20 h of *in vitro* challenge (performed in triplicate). Note: * *p<0*.*05*.

A previously established mouse calvarial model of osteolysis [41;42], was used to asses inflammatory bone loss caused by Cobalt-alloy particles +/- LPS implanted in WT (C57BL/6) 12 week old male mice over the calvarial surface. At 10 days post-treatment, calvaria were retrieved and analyzed for bone resorption by both histology and quantitative microCT. Histological evaluation (H&E staining) of the murine calvaria of tissue proximal to particle placement showed the potential of Cobalt particles to induce an inflammatory panus and the potential of this panus to invade the bone interface (Fig 6). Histological 2D analysis also showed that Cobalt-alloy/LPS+ resulted in the greatest inflammatory/osteolytic effect as measured by the thickness of the remaining peri-implantation calvarial bone, which was 54.85 um across in comparison to control bone at 193.8 um, Cobalt-alloy particles at 101.8 um (Fig 7). Quantitative uCT analysis demonstrated that Cobalt-alloy particles alone were sufficient to elicit a potent bone resorbing inflammatory tissue (Fig 8A and 8B).Moreover, TLR4 LPS at a high concentration alone (LPS at 5 μg/mL), had minimal effect and exhibited non-significantly increased bone resorption (compared to control, i.e. PBS only) in WT mice (Fig 8). In contrast, both Cobalt-alloy particles and Cobalt-alloy/LPS+ induced significantly greater bone resorption when compared to saline challenged control groups (Fig 8). Mice lack histidine pocket residues H456 and H458 in their TLR4 responsible for binding soluble Cobalt [17], thus, resultant inflammatory bone loss due to Cobalt particles alone were not attributable to TLR4 binding, and further demonstrated the dramatic potential of Cobalt alloy particles on the pathophysiology of inflammatory osteolysis independent of TLR4 activation. Although, Cobalt-alloy/LPS+ induced the most bone loss with 5 fold increase over controls (25% osteolysis), it was non-significantly greater compared to Cobalt-alloy alone (Fig 8), further mitigating the *in vivo* role of TLR4 in the pathophysiology of Cobalt induced osteolysis.

Similar results were also observed using murine peritoneal macrophages challenged with Cobalt-alloy particles (particles:cell = 5:1), Cobalt-alloy/LPS+ and LPS (100ng/mL) for 20 h using IL-1β and TNF-α production as outcome measures (Fig 8C). Cobalt-alloy particles significantly induced pro-inflammatory responses. Cobalt-alloy/LPS+ synergistically increased IL-1β compared to either Cobalt particles or LPS challenge alone but not TNF-α. Despite the previous contention that both Nickel and Cobalt ions do not activate murine TLR4 (due to lack in histidine residues), our data reveals that Cobalt alloy particles are independently capable of inducing a potent inflammatory and osteolytic effect and that the addition of LPS does not significantly augment this response compared to Cobalt particles alone. Therefore, the in vivo murine data indicates that TLR4 signaling pathway is not likely the dominant mechanism by which Cobalt alloy particles exert their proinflammatory effects, but rather it is NLRP3 inflammasome produced IL-1β, as IL-1β and not TNF-α was associated with inflammatory bone loss.

## Discussion

Our results indicate that Cobalt alloy implant debris alone or complexed with clinically extreme levels of the TLR4 ligand LPS, do not preferentially activate TLR4 induced inflammation compared to NLRP3 inflammasome danger signaling (IL-1β). Blocking TLR4 did not effectively decrease the inflammatory response of human monocytes/macrophages when challenged with Cobalt alloy, as would be expected if cobalt particles primarily induced TLR stimulation. Instead, blocking of the inflammasome pathway (via both Cathepsin B inhibitor and ZVAD-FMK) was highly effective in suppressing innate monocytes/macrophage inflammatory mediated responses to Cobalt alloy challenge. These responses to Cobalt alloy challenge differs from other types of implant debris (e.g. Polymers and Titanium) which have demonstrated TLR4 LPS has the capability to aggravate the inflammatory response when LPS dosed in vitro and in vivo at levels equivalent or greater than that associated with fatal sepsis [20;34;35;43]. But clinical applicability of using extremely high levels of endotoxin (commiserate or greater than that associated with sepsis) may not best indicate the primary mechanisms of inflammation at clinically more relevant levels of hematogenous endotoxin contamination.

Purportedly, to produce substantial amounts of IL-1β in vitro, monocytes/macrophages need to be primed with an initial PAMP stimulus, such as LPS, to generate accumulation of pro-IL-1β [44]. We and others have demonstrated that endotoxin free Cobalt and Titanium alloy metals can induce IL-1β release in the absence of PAMP-TLR stimulation. Therefore, metal debris as DAMP mediators alone, are sufficient to induce in vivo aseptic inflammatory responses and osteolysis [21;45;46]. Additionally, TLR4 LPS co-challenge failed to illicit significantly different responses from Cobalt alloy alone in our murine osteolysis model; further indicating Cobalt alloy as potent inflammatory activator independent of known mechanisms of Cobalt-TLR4 PAMPs binding.

Past investigation has shown that lysosomal destabilization is a critical process for Cobalt alloy activation of NLRP3 inflammasome [6]. Cobalt debris induces lysosomal damage with leakage of Cathepsin-B protease that leads to the activation of NLRP3 inflammasome and subsequent IL-1β secretion [46]. To clearly determine if this specific intracellular phenomenon has a dominant role in an immune response to Cobalt implant debris in combination with TLR4 ligand LPS, we inhibited a key lysosomal protease Cathepsin-B in human THP-1 macrophages and monocytes. We found blocking this lysosomal protease demonstrated significantly reduced IL-1β production to both challenge with Cobalt alloy alone and Cobalt alloy with LPS. Additionally, inhibiting the inflammasome pathway via ZVAD-FMK (inhibit caspase-1) was highly effective in mitigating Cobalt particle inflammasome mediated IL-1β. In contrast, TLR4 inhibition was non-effective in comparison to inflammasome specific blocking, given it did not significantly decrease IL-1β or TNF-α responses to Cobalt alloy challenge, in primary human monocytes/macrophages. That TNF-α production was increased by use of both ZVAD-FMK and PAb TLR4 with Cobalt alloy indicates that NLRP3 inflammasome and NFκB pathway are not mutually exclusive, and that there is complexity of cross-talk between the two signaling pathways and that more upstream blocking from NLRP3, such as inhibiting lysosomal Cathepsin B, more effectively decreases cross reactivity with the NFκB pathway and may be a potential strategy to mitigate particle induced inflammatory osteolysis.

IL-10 is a potent anti-inflammatory cytokine that limits excessive inflammation and promotes endotoxin LPS clearance in macrophages [47;48]. It has also been shown that chronic stimulation of both TLR2 and TLR4, induces IL-10 production that in turn negatively regulates NLRP3 inflammasome activation and mediated IL-1β secretion to circumvent overt inflammation[49]. Therefore, if Cobalt alloy induced TLR stimulation, then significantly increased levels of IL-10 secretion would also be expected, as was observed with LPS challenge. However, IL-10 was not significantly elevated in response to Cobalt alloy challenge. Collectively, these results further support our hypothesis that innate immune mediated inflammatory responses to Cobalt alloy is primarily danger signal mediated (NLRP3 inflammasome) when compared to TLR4 receptor activation.

Additionally, Cobalt alloy was able to induce extreme inflammatory bone loss *in vivo* despite the fact that murine TLR4 does not possess the histidine residues that have been previously reported to bind Nickel and Cobalt ions by human TLR4 [17;18;50]. And while Cobalt-alloy particles alone were able to induce inflammatory osteolysis in vivo, LPS alone was not. Micro-CT and histological analysis of the calvaria tissue proximal to Cobalt challenge alone or when combined with LPS, showed similar degrees of inflammatory bone loss and tissue invading the bone and causing osteolysis. These in vivo findings are consistent with our in vitro observations and indicate that Cobalt-alloy implant debris act as a highly potent danger signal acting through the inflammasome pathway to produce IL-1β and that this DAMP alone is sufficient to cause inflammatory osteolysis. Previous in vivo studies of polymeric particle induced inflammatory osteolysis, also indicated that DAMP induced danger signaling alone was able to induce inflammatory osteolysis, where Caspase-1 (critical to inflammasome activation) deficient mice demonstrated significantly less bone loss compared to wild type mice in response to calvaria challenge with endotoxin-free PMMA particles [45].

Our results only seemingly contrast with previous investigations of other types of metal implant debris [20;43;51], because adherent PAMPs to titanium particles (LPS) were observed to increase their biological reactivity in vitro and in vivo when investigated only from the perspective of one pathway, that of NFκΒ dependent TNF-α production. Additionally, previous investigations generally use *in vitro* doses of endotoxin that exceed serum levels associated with fatal sepsis (>500pg/mL), and may not optimally model the clinical process of inflammation to implant debris, where exposure to PAMPs, if any, is extremely low when there is no evidence of an infection (<2pg/mL) [34;35]. The lack of additional inflammatory reactivity (IL-1β or TNF-α or bone loss) when the TLR agonist LPS was combined with Cobalt alloy in our study at levels as high as that associated with hematogenous sepsis (>500pg/mL) [34;35] point to a minimal role for TLR activation in the pathogenesis of Cobalt alloy in orthopedic and non-orthopedic applications. This contention is supported clinically by previous reports of inherent genetic variations (i.e. polymorphisms) within the genes encoding IL-1 in people with more implant debris induced osteolysis [52;53]. These past studies together with our current data suggest the use of TLR inhibitors (or other downstream targets) may not be an effective pharmacologic means to mitigate implant debris reactivity in the presence or absence of subclinical levels of bacterial products to prevent/intervene with aseptic osteolysis and implant loosening.

**Fig 9:**
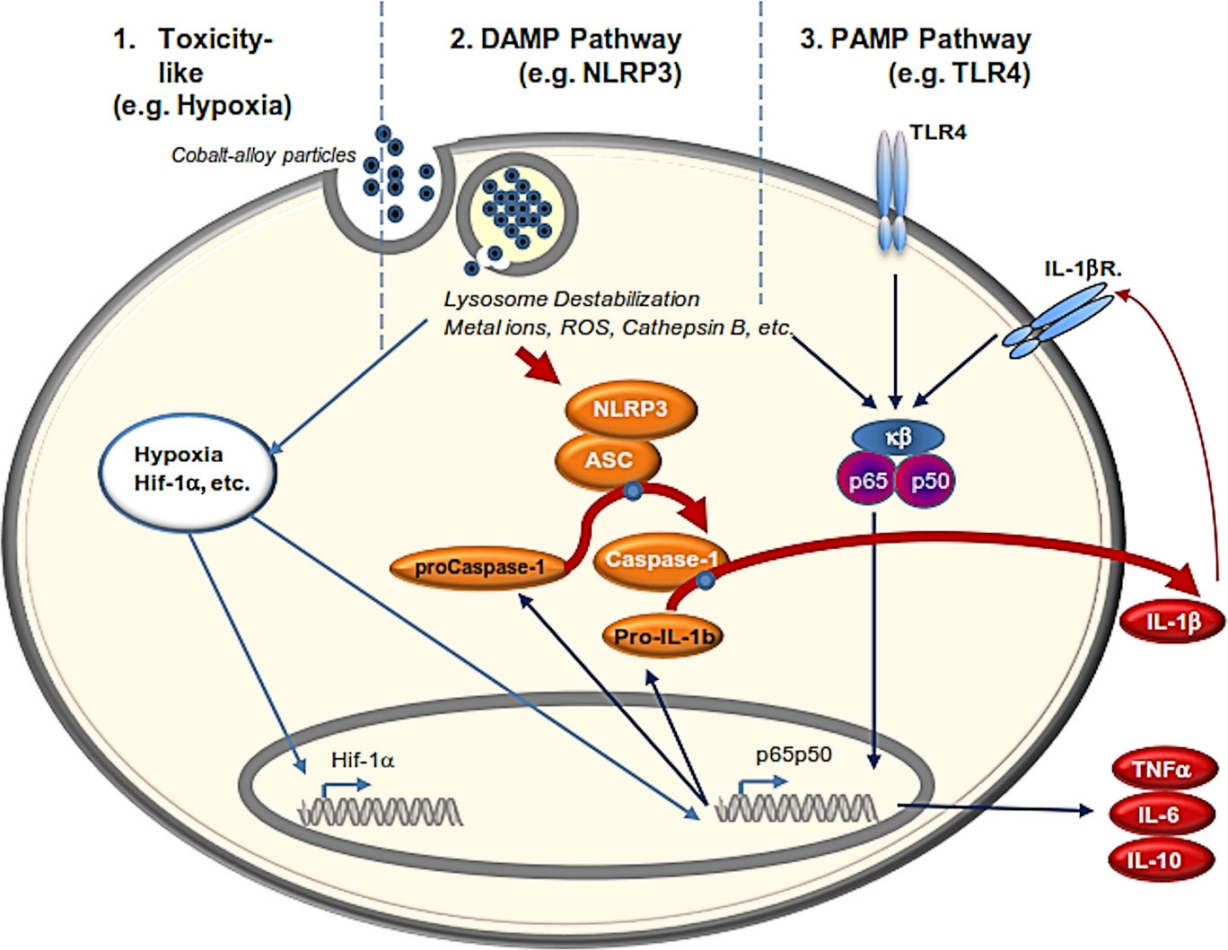
**A general schematic showing the effects of Cobalt alloy particulate on macrophages acting directly and indirectly on three major pro-inflammatory innate immune pathways**: 1) general toxins (such as inducing hypoxia like cell responses), 2) as danger associated molecular patterns, DAMPs (inflammasome induced activation), and 3) interacting with the pathogen associated molecular pattern (PAMP) pathway of TLR4.

It is unlikely that physiologic effects of metals (and toxins, stimulants etc in general) are limited to the actions of any single cellular pathway. And it is important to note the proliferation of pathway specific research may inappropriately reflect the reality of particular challenge agents, where the more dominant action and potency of many pathways must be compared to clearly assess likely physiologic consequences. The relative DAMP vs. PAMP effects of Cobalt alloy particles in this study may represent an example of this kind of comparative pathway research. The highly pleiotropic nature of Cobalt binding to different proteins and the myriad effects of cobalt on macrophages, may be summarized as DAMPS, PAMPs and as general toxins (such as inducing hypoxia like cell responses), Fig 9. Evidence for all three types of reactivity has been reported. We have previously demonstrated that Cobalt alloy is a potent stimulus of inducing a hypoxic microenvironment that results in HIF-1α accumulation and stabilization [3]. We have also previously reported the relative genotoxicity of cobalt (and other orthopedic metals) on bone and immune cells and found that genotoxicity can occur but generally only at levels that are extremely toxic and have induced greater than 50% cell necrosis [14;54]. It has also been demonstrated that that Nickel and Cobalt ions can induce IL-8 production via human TLR4 activation [17;55]. However, it has also been reported that hypoxia can result in up-regulation of both TLR4 expression and IL-8 production and thus, whether this TLR4 induced IL-8 is a primary or secondary effect of Cobalt is not known [56;57]. Therefore, it is possible that previous investigations of observed cobalt induced IL-8 may have been due to Cobalt toxicity responses eliciting a hypoxia state and increased endogenous alarmins/DAMPs (i.e. HMGB1, HSP60), rather than direct TLR4 activation. This underscores the need to assess metal (and other) challenge agents relative activation of different pathways such as the DAMP vs. PAMP pathways involved in innate immune reactivity. *In vitro* and *in vivo* Cobalt alloy bioreactivity in the current study was shown strong enough to illicit danger signaling pro-inflammatory responses that dominated any concomitant TLR activation at concentrations of metal that were not toxic (>90% viability but with the likelihood of additionally induced stress signals, e.g. HIF-1α). This unique DAMP response profile of Cobalt alloy (or its degradation products) may explain the unique unexpected pathogenicity of some current metal on metal articulating orthopedic implants. This cobalt specific bioreactivity has important implications for mediating both orthopedic and generalized forms of Cobalt pathogenecity. Alternatively, this unique Cobalt-specific reactivity may have possible positive applications where strong danger signaling is desired in such applications as vaccine adjuvant (e.g. as a mercury substitute) and immunostimulation modalities for cancer therapy. However, this may be overly risky given the possibility of autoimmune and adaptive immune responses (metal hypersensitivity) and cobalt related organ-specific pathologies.

In summary, our in vitro and in vivo data indicate that Cobalt alloy particles induce macrophage inflammation and that blocking danger signaling completely abrogated these responses, whereas attempts to block TLR4 had little to no effect and that *in vivo* responses in mice lacking histidine residues for binding Cobalt, demonstrated TLR4 independent extreme inflammatory bone loss. High doses of the TLR4 agonist LPS combined with Cobalt-alloy particles did act synergistically in differentiated primary human macrophages, to produce high levels of IL-1β, TNF-α, IL-6 and IL-10, while Cobalt alone failed to induce IL-10 responses, indicating the synergistic interaction of the PAMP vs. DAMP pathways (Fig 9). In vivo cobalt alone independent of TLR4 induce extreme inflammatory bone loss and the addition of LPS did not significantly contribute to this response, despite the use of TLR agonists (LPS) at levels higher than those found systemically in fatal sepsis [34;35]. Orthopedic implant failures due to excessive innate immune reactivity to Cobalt debris are likely inflammasome driven and may be the result of direct and secondary DAMPs as well as other specific toxicity responses (e.g. hypoxia[3]) but are not likely dominated by TLR4 involvement/activation.
